# Acute ST-Elevation Myocardial Infarction Presenting With Persistent Vomiting

**DOI:** 10.7759/cureus.26920

**Published:** 2022-07-16

**Authors:** Zoheb Backer, Bola Nashed, Arshan A Khan, Mohamed Issa, Krishna Mahat

**Affiliations:** 1 Internal Medicine, Ascension St. John Hospital, Detroit, USA

**Keywords:** st-elevation myocardial infarction (stemi), ekg abnormalities, nausea and vomiting, diabetes type 2, acute coronary syndrome (acs) and stemi

## Abstract

Atypical presentations of acute coronary syndrome (ACS) have been commonly known to occur but are often excluded in the differential when other diagnoses seem more likely. Female gender, patients with diabetes, hypertension, age greater than 55, and a history of smoking are some of the risk factors that have been associated with noncharacteristic presentations of ACS. This often leads to misdiagnosis and overall increased mortality. Patients with risk factors for atypical presentations of myocardial infarctions should mandate a low threshold for suspicion and undergo evaluation with EKG and troponins for prompt diagnosis and early intervention.

## Introduction

Acute coronary syndrome (ACS) presenting with atypical symptoms is more frequently seen in elderly patients aged 55 or older and those with multiple comorbidities, especially for patients with diabetes [1-2]. Women are another subgroup with a higher likelihood to have atypical presentation patterns. Atypical presentations include the absence of chest pain and the presence of nonspecific symptoms such as nausea or vomiting, sweating, dyspnea or cough, and non-chest pain localized to the neck, back, jaw, or head [3]. The etiology of symptomatology appears to be multifactorial, with the presence of traditional risk factors along with the extent of myocardial infarction possibly influencing symptoms. The site of infarction also appears to be linked to specific groups of symptoms [4]. Here, we report the case of a 54-year-old male who presented with severe vomiting and no history of chest pain and was diagnosed with an acute ST-elevation myocardial infarction (STEMI) on a routine 12-lead electrocardiogram (ECG) following which he was taken for emergent percutaneous coronary intervention (PCI).

## Case presentation

A 54-year-old male patient with no previous documented medical history presented to the emergency room (ER) with complaints of severe persistent vomiting, approximately 30-40 episodes of sudden onset about 20 hours prior. He also complained of a mild epigastric burning sensation prior to the onset of vomiting. He denied previous similar symptoms, chest pain, palpitations, lightheadedness, or shortness of breath. He had no previous history of diabetes mellitus, hypertension, hyperlipidemia, or a family history of coronary artery disease. He had a 10-pack-year history of smoking.

The initial evaluation in the ER showed a clinically dehydrated patient who was hemodynamically stable with a temperature of 98.6° Fahrenheit (F), heart rate of 95 beats per minute, respiratory rate of 20 breaths per minute, oxygen saturation of 99%, and blood pressure of 130/88 mmHg. He was conscious, alert, and in no apparent distress. Physical examination showed no abnormalities except for a dry coated tongue. An abdominal exam showed no tenderness, rigidity, guarding, or organomegaly. Bowel sounds were normal. Cardiovascular examination disclosed normal S1, and S2 with no murmurs, gallops, or rub. The pulmonary exam was normal with bilateral equal breath sounds and no rhonchi or rales.

Initial investigations were significant for neutrophilic leukocytosis with a total count of 22,770 and elevated random blood sugar of 391 mg/dl. Urine analysis showed 4+ ketones and 4+ glucose. Venous blood gas analysis showed a pH of 7.4 with bicarbonate of 25. He also had transaminitis with elevated AST. He had a normal lipase. The differential diagnosis considered at this time included gastritis, hepatitis, and diabetic ketosis without acidosis. Initial treatment in the ER was aimed at correcting dehydration, hyperglycemia from uncontrolled newly diagnosed diabetes, and symptomatic management of vomiting. Diabetic ketoacidosis (DKA) protocol was not initiated as the patient was not acidotic. The patient was initiated on normal saline (NS) bolus, and regular insulin was administered subcutaneously using a sliding scale, ondansetron, and pantoprazole (Table 1).

**Table 1:** Laboratory values AST: aspartate transaminase, ALT: alanine transaminase, CRP: C-reactive protein, PO_2_: partial pressure of oxygen, PCO_2_: partial pressure of carbon dioxide, HCO_3_: bicarbonate, pro-BNP: pro-B-type natriuretic peptide.

Lab	Patient value	Normal value
Troponin	3.460	0-0.014 ng/ml
Pro-BNP	1742.000	<75 years: <125 pg/ml; >75 years: <450 pg/ml
Total leukocyte count	22.7	4-11 x10^3/L
Neutrophils	87.7	40%-80%
Hemoglobin	15.2	13-17 g/dl
Platelets	245	150-400 x10^3/L
AST	262.2	0-36 U/L
ALT	49.1	0-50 U/L
CRP	13	0-36
Glucose	391	70-140 mg/dl
Sodium	135.8	132-146 mmol/L
Potassium	4.2	132-146 mmol/L
Chloride	93	98-106 mmol/L
Lipase	62	10-140 U/L
Urine ketones	4+	Absent
Urine glucose	4+	Negative
Urine protein	1+	Absent
Urine blood	1+	Absent
Venous blood gas		
pH	7.4	7.30-7.45
PO_2_	39	23-47 mmHg
PCO_2_	46	40-54 mmHg
HCO_3_	25	16-28 mmol/L

**Figure 1:**
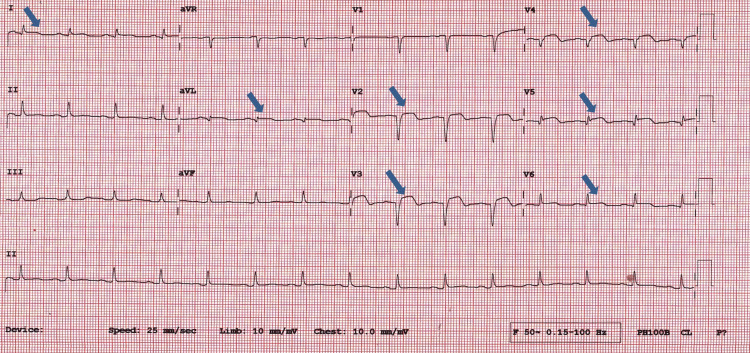
ST elevations seen on ECG in leads I, aVL, V2-V6 aVR: augmented vector right, aVL: augmented vector left, aVF: augmented vector foot, P: P wave, CL: cycle length.

He was admitted to the floor for supportive therapy with a continuous infusion of IV fluids and antiemetics as needed. Due to the patient's brief history of epigastric pain and history of smoking, an EKG felt warranted and was taken during physician rounds which showed a fully evolved and extensive anterolateral STEMI with 1-2 mm ST elevation in leads I, augmented vector left (aVL), V2 through V6 (Figure 1). Cardiac troponins and pro-B-type natriuretic peptide (pro-BNP) ordered showed elevated cardiac troponin of 3.46 ng/ml (N<0.014) and pro-BNP of 1,742 pg/ml. ACS protocol was initiated with aspirin, clopidogrel, bisoprolol, ramipril, and low-molecular-weight heparin (LMWH). Chest X-ray showed pulmonary congestion in view of which NS was stopped, and furosemide was administered. A cardiology referral was made, and a bedside echo performed demonstrated an ejection fraction (EF) of approximately 40% with global hypokinesis. The patient was immediately transferred to a center with interventional facilities available for urgent coronary angiography, which showed a 99% occlusion in mid-left anterior descending artery (LAD), a 90% occlusion in distal LAD, and a 90% occlusion in posterior descending artery (PAD) branch of the right coronary artery (RCA). PCI was performed in which three drug-eluting stents were placed. The patient tolerated the procedure well with no adverse events. The patient was discharged without any complications 24 hours after the intervention.

## Discussion

This report serves to emphasize the detrimental effects of overlooking cardiovascular causes in patients presenting with vomiting and epigastric discomfort. These presentations are known to occur but are frequently misdiagnosed especially in smaller centers with young inexperienced physicians and in patients who do not have the risk factors associated with atypical presentations of ACS including no history of diabetes, younger age, and male gender. ACS is a common and potentially life-threatening condition encountered at the ER [5]. Despite its dreaded nature, ACS may frequently mislead clinicians with atypical presentations, presenting without chest pain (33%) and with dominant symptoms of dyspnea (38%), nausea, and/or vomiting (26%) [6-7]. Increased mortality was also noted in patients with presenting symptoms of presyncope/syncope (odds ratio [OR], 2.0; 95% confidence interval [CI], 1.4 to 2.9), nausea/vomiting (OR, 1.6; 95% CI, 1.1 to 2.4), and dyspnea (OR, 1.4; 95% CI, 1.1 to 1.9) [8]. The atypical symptoms tend to occur more commonly among those who are older (age >55 years), female, diabetic (possibly due to autonomic neuropathy), hypertensive, and with a history of prior heart failure [5]. Patients without chest pain on presentation represent a large segment of the ACS population and are at increased risk for delays in seeking medical attention, less aggressive treatments, and in-hospital mortality [7].

Although a relatively straightforward case could have been diagnosed with a routine EKG, atypical presentations of ACS are frequently missed in the ER which, in our case, was likely due to the patient's presentation with no past medical history, not having the risk factors associated with atypical presentations of ACS, and symptoms and laboratory results suggesting alternative diagnoses to be considered. Due to the delay in establishing the diagnosis of STEMI, our patient was at an increased risk for morbidity and mortality. Admission to the floor without telemetry along with potentially overloading him with IV fluids for treatment of dehydration and a new diagnosis of diabetes served to increase his risk of developing avoidable complications such as cardiac arrhythmias or overt cardiac failure. Had the diagnosis been established earlier, he could have been promptly considered for medical thrombolysis or referral to a higher center for PCI substantially decreasing his risk of mortality.

## Conclusions

In conclusion, the varying nature of the presentation of ACS can be a diagnostic challenge to clinicians and hence mandates a low threshold for suspicion. It is imperative for adequate cardiovascular workup to be performed in the ER even in the absence of typical symptoms and significant cardiovascular risk factors.
